# On the Origin and Trigger of the Notothenioid Adaptive Radiation

**DOI:** 10.1371/journal.pone.0018911

**Published:** 2011-04-18

**Authors:** Michael Matschiner, Reinhold Hanel, Walter Salzburger

**Affiliations:** 1 Zoological Institute, University of Basel, Basel, Switzerland; 2 Institute of Fisheries Ecology, Johann Heinrich von Thünen-Institute, Federal Research Institute for Rural Areas, Forestry and Fisheries, Hamburg, Germany; Laboratoire Arago, France

## Abstract

Adaptive radiation is usually triggered by ecological opportunity, arising through (*i*) the colonization of a new habitat by its progenitor; (*ii*) the extinction of competitors; or (*iii*) the emergence of an evolutionary key innovation in the ancestral lineage. Support for the key innovation hypothesis is scarce, however, even in textbook examples of adaptive radiation. Antifreeze glycoproteins (AFGPs) have been proposed as putative key innovation for the adaptive radiation of notothenioid fishes in the ice-cold waters of Antarctica. A crucial prerequisite for this assumption is the concurrence of the notothenioid radiation with the onset of Antarctic sea ice conditions. Here, we use a fossil-calibrated multi-marker phylogeny of nothothenioid and related acanthomorph fishes to date AFGP emergence and the notothenioid radiation. All time-constraints are cross-validated to assess their reliability resulting in six powerful calibration points. We find that the notothenioid radiation began near the Oligocene-Miocene transition, which coincides with the increasing presence of Antarctic sea ice. Divergence dates of notothenioids are thus consistent with the key innovation hypothesis of AFGP. Early notothenioid divergences are furthermore congruent with vicariant speciation and the breakup of Gondwana.

## Introduction

Adaptive radiation - the evolution of ecological and phenotypic diversity within a rapidly multiplying lineage - has been implicated in the genesis of a great portion of the diversity of life [1], [2]. According to Schluter [2], an adaptive radiation is characterized by rapid speciation, common ancestry, and a phenotype-environment correlation, whereby phenotypes must actually be beneficial in their respective environments. Adaptive radiation is often considered a consequence of ecological opportunity [1], [2] arising through colonization of a new habitat with abundant niche-space, extinction of antagonists, and/or the origin of a key innovation [3]. All three settings induce the relaxation of selection pressure, which may promote diversification [3]. Key innovations can lead to ecological opportunity either by enabling the exploitation of new resources, or by boosting a clade's fitness relative to competing lineages. A third type of key innovation does not generate ecological opportunity, but directly enhances diversification rates by increasing the potential for reproductive isolation or ecological specialization, e.g. by decreasing dispersal distance and gene flow [4]. Corroboration of the key innovation hypothesis would, hence, involve the identification of (ecological) mechanisms linking a putative key innovation to increased speciation or decreased extinction rates, and comparative tests correlating it with inflating diversity [4].

So far, such tests have been applied to few key innovations only, and even the best examples of animal adaptive radiations provide only scanty evidence in support of the key innovation hypothesis. Two of the most prominent examples of adaptive radiation, the Galapagos finches and Hawaiian honeycreepers, were, in fact, more likely triggered by the arrival of the ancestral species on competitor-free islands rather than by key innovations [5]. A number of key innovations have been proposed for the radiations of cichlid fishes in the Great Lakes of East Africa, including a highly variable pharyngeal jaw apparatus, egg-spots, and maternal mouth brooding behaviour [6], [7]. However, based on a comparative analysis of successful and failed cichlid radiations, the role of all three traits as key innovations has been questioned [8]. Similarly, the acquisition of pharyngeal jaws provides a weak explanation for increased diversification rates in the radiation of labrid fishes [9]. A key innovation in Caribbean *Anolis* lizards, on the other hand, appears to pass both the ecological mechanism and comparative test: extended subdigital toe-pads enable *Anolis* to climb narrow twigs, leaves and grass blades. The resultant arborality distinguishes them from other iguanids. Toepads evolved at the base of the anole phylogeny, and also occur in the second-most species rich family of lizards, the Gekkonidae, thus linking its emergence with species richness [10].

Another vertebrate adaptive radiation, which has drawn increasing interest in recent years, has occurred on the isolated shelf areas surrounding the Antarctic continent in the perciform fish suborder Notothenioidei. A total of 132 notothenioid species are known to date, and new species are discovered at fast rates [11]. Nine species belong to three early diverging families (Bovichtidae, Pseudaphritidae, Eleginopidae) that occur almost exclusively outside Antarctic waters and are not usually considered part of the radiation. The remaining 123 species in five families are often referred to as the “Antarctic Clade”, which dominates the High Antarctic ichthyofauna in terms of species number (76.6%) and biomass (>90%) [11]. Notothenioids of the Antarctic Clade possess a wide range of adaptations to the extreme Antarctic environment, including antifreeze glycoproteins (AFGP) [12], retinal reorganization [13], and loss of heat shock response [14]. One of the five Antarctic notothenioid families (Channichthyidae) even lives without hemoglobin, which is unique among vertebrates [15]. Despite the loss of the swim bladder in their presumably benthos-dwelling ancestor, multiple notothenioid lineages have independently recolonized pelagic, semi-pelagic and cryopelagic habitats. Subsequent adaptations in ossification, scale mineralization, and lipid deposition led to a partial or full reaquisition of neutral buoyancy and a phenotype-environment correlation that is characteristic for adaptive radiations [2], [11]. Another important phenotype-environment correlation exists between freezing avoidance and water temperature [16].

Antifreeze glycoproteins are present in almost all notothenioids of the Antarctic Clade, enabling them to cope with the subzero temperatures of Antarctic waters [17]. The widespread possession of AFGPs in the monophyletic Antarctic Clade, complete lack of AFGPs in non-Antarctic sister groups, and their highly conserved chemical structure [15] suggest that AFGPs evolved only once in notothenioids and that this occurred prior to the onset of diversification in the Antarctic Clade [12], [17]. Therefore, it has been hypothesized that AFGPs represent a key innovation that allowed notothenioids to radiate at a time when Antarctic water temperatures dropped below zero, which presumably led to the extinction of a great part of the previous Antarctic shelf ichthyofauna [15]. Following Heard and Hauser [4] AFGPs would constitute a type I key innovation if the resulting fitness advantage enabled notothenioids to replace other clades, a type II key innovation if AFGPs allowed the invasion of previously unoccupied sea ice-associated habitats, or a combination of both. A crucial prerequisite for either hypothesis is the concurrence of the beginning of the notothenioid radiation and the onset of Antarctic sea ice conditions.

Cenozoic Antarctic water temperatures and the emergence of sea-ice in Antarctica can be inferred from deep sea isotope records and sediment analysis of drill cores [18]–[21]. The timing of the notothenioid radiation, on the other hand, is far less certain, which is in part due to the paucity of fossils in Antarctica. Existing molecular clock calibrations for notothenioids are based on few mitochondrial markers in combination with a single putative, but debated, eleginopid fossil [22], biogeographic patterns [23], or the presumed date of the perciform diversification [24]. Consequently, attempts to date the beginning of the notothenioid radiation have led to a wide range of contradicting results between 7 and 24 Ma [22], [25].

Here we use a multi-marker (4599 bp, 6.53% missing data) phylogeny including representatives of all notothenioid families plus 69 non-notothenioid fishes with ten fossil and phylogeographic constraints to time-calibrate notothenioid divergences and AFGP evolution.

## Results

### Tree Topology

**Figure 1:**
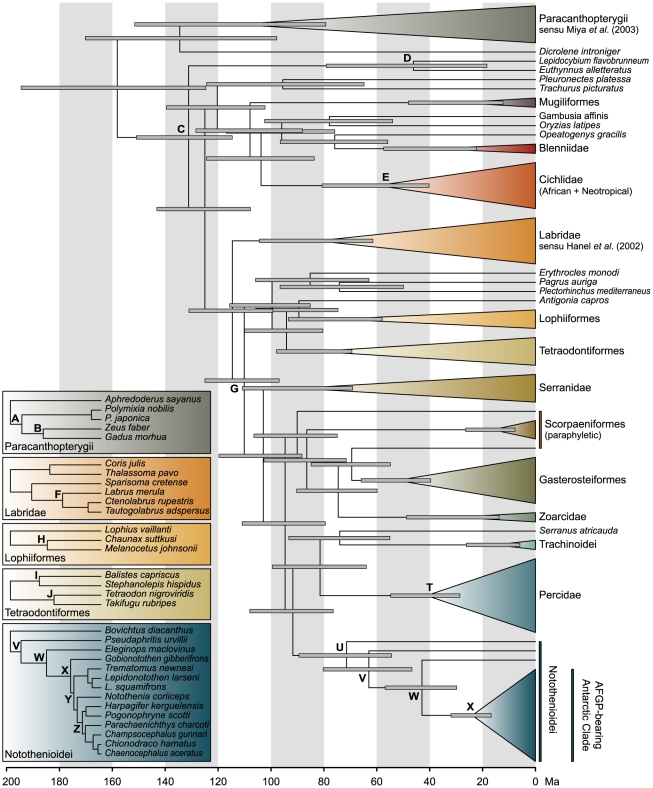
Time-calibrated phylogeny of acanthomorph fishes based on the concatenated dataset of two mitochondrial and four nuclear genes, and six calibration points (B, C, G, H, I, and J). All nodes used for constraint cross-validation are labelled with letters A–J, percid and notothenioid nodes are labelled with letters V–Z. Insets indicate nodes labels within Paracanthopterygii, Labridae, Lophiiformes, and Tetraodontiformes. Node bars show 95% HPD.

Partitioned Maximum Likelihood and Bayesian phylogenetic analyses of 83 acanthomorph taxa using GARLI-PART, RAxML, and BEAST (Fig. 1, Fig. S2) resulted in identical topologies with the exception of the position of *Antigonia capros*, which was placed as sister group to Lophiiformes and Tetraodontiformes in RAxML's optimal tree. The phylogenetic placement of zeioids within Paracanthopterygii [26] and scarids within Labridae [27], [28] was confirmed in all analyses. Gasterosteiformes and Zoarcidae appeared within Scorpaeniformes, thus rendering this order paraphyletic (albeit with low support values). Notothenioids were recovered as a sister group of a clade containing percids, trachinoids, and *Serranus atricauda*. The highly supported placement of the latter (Bayesian Posterior Probability (BPP) 1.0; Fig. S2, Table S1) is in accordance with previous phylogenetic hypotheses and suggests polyphyly of serranids, with representatives of the family in close phylogenetic affiliation with notothenioids, percids, and trachinoids [29], [30]. Removal of *S. atricauda* from the data set affected the tree topology only in the weakly supported position of *Antigonia capros* (Table S1). The notothenioids were covered by 14 (phylogenetically) representative species. While this number might appear small in the context of notothenioid phylogenetics and divergence rate estimates (which was, notably, not the purpose of this study), it is absolutely balanced with respect to the timing of their radiation and their relative coverage in the total dataset. Our trees confirm the divergence of Bovichtidae prior to Pseudaphritidae, the monophyly of the Antarctic Clade, the paraphyly of the family Nototheniidae within the Antarctic Clade, and the interrelationships of derived notothenioid families (Fig. S2) [22], [31].

### Cross-Validation of Time Constraints

**Figure 2:**
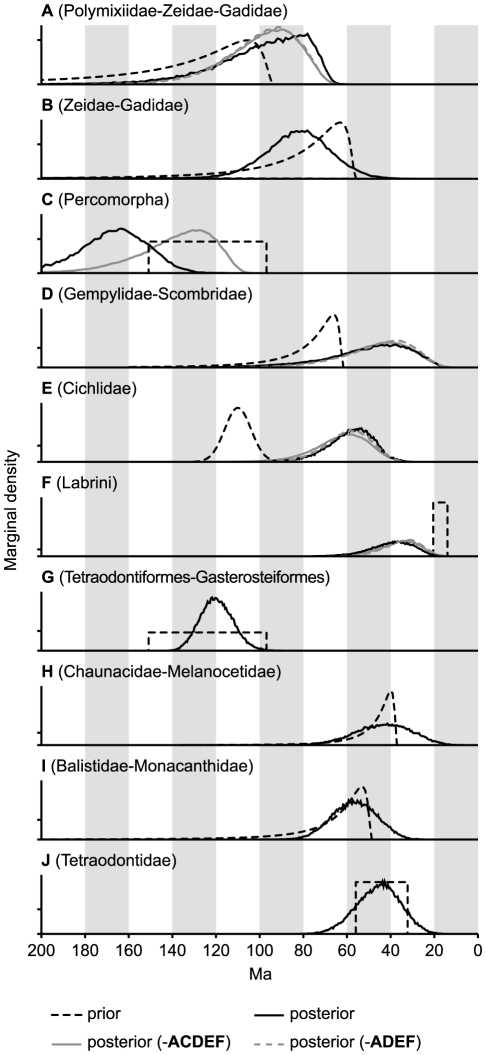
Cross validation of all fossil and phylogeographic constraints. The dashed black line indicates the prior, as specified in all BEAST runs including this constraint. The solid black line shows the marginal densities of BPPs for each node when its constraint is relaxed and its date estimated based on all other constraints. At this stage, only five out of ten (B, G, H, I, J) nodes showed a good fit between prior and posterior. A new BEAST analysis (‘-ACDEF’) was conducted, using only constraints B, G, H, I, and J. Results of this analysis (solid grey line) showed a good fit between prior and posterior for node C, therefore node C was reincluded in a final BEAST run (‘-ADEF’; dashed grey line).

We first cross-validated the available 10 calibration points in order to test for their relative consistency. This step is important, as calibrations based on fossil and geological data show various degrees of uncertainty [32]. When estimated on the basis of all other constraints, five out of ten divergence dates were concordant with the respective fossil age assignments (Fig. 2). The split between gempylids and scombrids (node D; 79.2–18.4 Ma, 95% highest probability density (HPD)) seems to postdate respective fossil findings and suggest taxonomic or stratigraphic misinterpretations. The mean age estimate for the polymixiid lineage (99.2 Ma) falls into the Cenomanian, as does the oldest polymixiid fossil. Nevertheless, this constraint was excluded from further analyses, as nearly half of its HPD (133.1–70.5 Ma) postdates the Cenomanian. Age estimates for both cichlid and labrid divergences failed to match phylogeographic calibrations, which were therefore excluded. Estimated on the basis of nine constraints, the diversification of Percomorpha (203.3–135.0 Ma) seems to predate the earliest euteleost fossils (150.9 Ma) [33]. However, after exclusion of constraints A, C, D, E, and F, re-estimation resulted in younger percomorph divergence estimates (150.9–114.7 Ma), being congruent with the euteleost fossil record.

### Notothenioid Divergence Dates

**Figure 3:**
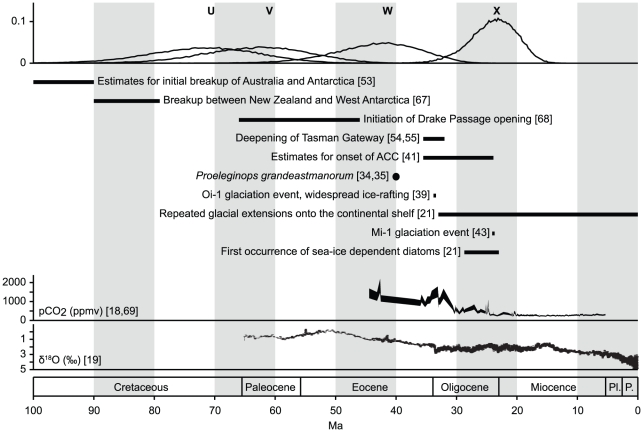
Comparison of notothenioid divergence dates and geological events. Phylogenetic nodes are labelled with letters U-X according to Fig. 1. The diversification of the Antarctic Clade (node X) coincides with an increase in frequency of glacial extensions well onto the shelf, the Mi-1 glacial event, the first occurences of sea-ice dependent diatoms in Antarctic waters, and a sharp decline of atmospheric CO_2_ levels. Paleoc.:Paleocene, Oligoc.:Oligocene, Pl.:Pliocene, P.:Pleistocene.

According to cross-validation results, we estimated divergence dates of notothenioids on the basis of six fossil constraints (nodes B, C, G, H, I, and J; Fig. 3, Table S2). Our results support a late Cretaceous origin of Bovichtidae (node U, mean 71.4 Ma, 95% HPD 89.4–54.4 Ma), early Paleocene divergence of Pseudaphritidae (node V, 63.0 Ma, 80.2–46.7 Ma), and a Mid-Eocene split between Eleginopidae and the Antarctic Clade (node W, 42.9 Ma, 56.9–29.8 Ma). Mid-Eocene origin of the eleginopid lineage is congruent with the age of the only fossil putatively assigned to Notothenioidei. *Proeleginops grandeastmanorum* from the La Meseta Formation on Seymour Island (dated to ∼40 Ma) was first described as a gadiform fossil [34], and subsequently reinterpreted as an eleginopid [35]. While previous attempts to date the notothenioid radiation used this fossil as a single calibration point, our analysis deliberately excluded this constraint due to its debated taxonomic assignment [22]. Nevertheless, the concordance between divergence date estimates and fossil age corroborates the eleginopid interpretation of *P. grandeastmanorum*. Radiation of the Antarctic Clade began near the Oligocene-Miocene transition with the divergence of *Gobionotothen* (node X, 23.9 Ma, 31.9–16.7 Ma) and was quickly followed by further diversification within the Antarctic Clade (node Y, 21.4 Ma, 28.2–15.3 Ma). Correspondingly, support values for early nototheniid divergences are low (node Y, Table S1), indicating rapid succession of speciation events. Excluding *G. gibberifrons* from the data set indicates that regardless of the exact topology of the Antarctic Clade, the radiation was underway in the early Miocene (23.0 Ma, 30.5–16.1 Ma). This is in agreement with previous age estimates (24.1±0.5 Ma) on the basis of the putative eleginopid fossil *P. grandeastmanorum* and a penalized likelihood molecular analysis approach [22]. Diversification of the four most derived notothenioid families Harpagiferidae, Artedidraconidae, Bathydraconidae, and Channichthyidae apparently began in the mid-Miocene (node Z, 14.7 Ma, 20.0–9.9 Ma). Close agreement of percid divergence dates (node T, 28.54–54.71 Ma) with biogeographical scenarios suggests that clades closely related to notothenioids were dated reliably (Text S1). All acanthomorph divergence date estimates are summarized in Table S2. Notothenioid divergence date estimates are relatively robust to the set of constraints used for our cross-validation (Table S3).

## Discussion

### Antifreeze Glycoproteins are a Key Innovation

Bayesian Inference of acanthomorph divergence dates shows that the adaptive radiation of Antarctic notothenioids, resulting in more than 120 morphologically highly diverse species that dominate Antarctic waters, began near the Oligocene-Miocene transition (mean 23.9 Ma, 95% HPD 31.9–16.7 Ma). While large-scale continental glaciation may not have been permanent before the middle Miocene climate transition (∼14 Ma) [36], [37], geological evidence supports temporal presence of Antarctic sea ice already 24 Ma: Deep-sea oxygen (δ^18^O) isotopes (Fig. 3) provide a reliable record of relative temperature changes and demonstrate an overall cooling trend (∼14°C) since the early Eocene [19]. Similarly, isotope levels of sedimentary alkenones reveal partial pressures of paleoatmospheric carbon dioxide (*p*CO_2_; Fig. 3) and show a decrease from the middle to late Eocene that led to rapid expansions of large continental Antarctic ice sheets and widespread ice rafting as early as 34 Ma [18], [38], [39]. Numerical climate models with explicit, dynamical representations of sea ice show that moderate or full cenozoic glaciation of East Antarctica would have promoted extensive sea ice formation at least in cold austral summer orbits with low *p*CO_2_ levels (560 ppmv) [40]. Direct evidence for continental glaciation and marine ice comes from cyclic glacimarine deposits in offshore drill cores, showing that glacial extensions well onto the continental shelf occurred repeatedly since the early Oligocene (Fig. 3) [20]. Furthermore, the long-term presence of local sea ice is suggested by findings of sea ice-dependent diatoms in lower Oligocene sediments [21]. Taken together, it is likely that Antarctic sea ice has existed with seasonal, orbital, and local constraints since the early Oligocene. Estimates for the onset of the Antarctic Circumpolar Current range widely [41], but its increasing strength presumably contributed to thermal isolation and cooling of Antarctic waters by up to 4°C during the Oligocene and Miocene [42]. Deep sea oxygen isotope records and glacimarine sediments further indicate a major period of global cooling and ice sheet expansion at the Oligocene-Miocene transition (Mi-1 event, 24.1–23.7 Ma, Fig. 3) [43]. This exactly coincides with our mean age estimate for the onset of the Antarctic notothenioid radiation (23.9 Ma), which is characterized by the presence of AFGPs. Based on the highly conserved chemical structure of AFGPs in nearly all notothenioids of the Antarctic Clade [15], it is commonly assumed that AFGPs evolved only once, before the notothenioid radiation [12], [17]. Members of the genus *Patagonotothen* apparently lack AFGPs [17], which is – however – likely due to secondary loss (as *Patagonotothen* is deeply nested within AFGP-bearing nototheniids, it would otherwise require at least 6 independent origins of notothenioid AFGPs [15], [31]). Hence, the key innovation hypothesis of AFGP is consistent with our age estimate for the notothenioid radiation. Freezing avoidance could have allowed notothenioids to invade new, ice-associated niches, or to replace other clades subsequent to their extinction in a cooling environment. Without doubt, selection pressures must have been substantial, given that freezing avoidance is a matter of life and death in ice-laden habitats [44]. Emergence of AFGPs could have proceeded in a step-wise manner that began with accidental replication slippage in an intron of the ancestral trypsinogen gene [12]. Subsequent duplications of Thr-Ala-Ala tripeptides could have endowed some measures of freezing avoidance without immediate loss of trypsin activity [44].

The key innovation hypothesis of AFGP would further be corroborated if similar diversity was found in other clades that independently acquired freezing avoidance [4]. Outside notothenioids, near-identical AFGPs have convergently evolved in Arctic cod *Boreogadus saida* [45] and other taxa of the subfamily Gadinae [46], [47]. Apparently, cod AFGPs share a common origin that dates back to the Miocene [48]. The subfamily consists of 23 species and may thus be considered moderately species-rich. Type III antifreeze proteins (AFP) are found in zoarcids of both Antarctic and Arctic waters, and supposedly predate the bipolar distribution of zoarcids [47], [49]. With over 200 species, the family Zoarcidae is indeed a highly diverse group [49], and surpasses even notothenioids in species richness. However, AFPs have been identified in comparatively few zoarcids to date [47], which may indicate secondary losses in many taxa. Whether AFP played a role in the zoarcid radiation remains to be elucidated. The distribution of type I AFPs over phylogenetically distant clupeids, osmerids, and cottids of the northern hemisphere provides a rare example of lateral gene flow in vertebrates [50] and indicates strong selection pressures. However, none of these AFP-bearing taxa have undergone substantial radiations. This could be due to external factors that mask the effect of AF(G)P emergence in non-notothenioid taxa. Most adaptive radiations occur in geographically confined areas, a potential prerequisite [51] that is satisfied for Antarctic continental shelves, but less so for Arctic habitats. In addition, dispersal of zoarcids to Antarctica in the Miocene [49], when the notothenioid diversification had already filled most available niche space, could have limited their radiation.

### Phylogeography of Notothenioid Lineages

Estimates of early notothenioid divergence dates support most aspects of the phylogeographic scenario proposed by Balushkin [52]: The presence or even endemism of three out of four bovichtid and pseudaphritid genera in Australia suggests occurrence of the presumably benthic notothenioid ancestor [15] on South Australian continental shelves in the late Cretaceous. Fragmentation of shelf areas between Australia and New Zealand ∼70 Ma may have led to the separation of bovichtids from the pseudaphritid ancestor and to initial divergences within Bovichtidae. Extended pelagic larval durations could have contributed to long-ranged eastward dispersal of bovichtids with paleogene currents to South America and Tristan da Cunha [52]. The isolation of pseudaphritids in Southern Australia and divergence of the Antarctic lineage are presumably linked to the breakup of Australia and Antarctica. Separation between both landmasses started between 125 and 90 Ma [53], however, shallow water connections existed until ∼33.5 Ma [54], [55]. Vicariant speciation of benthic lineages could have occurred anytime between these dates, being supported by our age estimate of pseudaphritids (node V, 63.0 Ma, 80.3−46.7 Ma). Antarctic notothenioids then diversified into eleginopids and the ancestor of the Antarctic Clade in the Eocene (node W, 42.9 Ma, 56.9−29.8 Ma). Past presence of eleginopids in Antarctica is indicated by the fossil *P. grandeastmanorum*, presumably representing an early member of the lineage [35]. Finally, a drop in water temperatures and the increasing presence of sea ice in the Oligocene led to near-complete replacement of the Eocene Antarctic ichthyofauna [15], migration of eleginopids to South America, and adaptive radiation of the Antarctic Clade subsequent to AFGP emergence.

## Materials and Methods

### Phylogenetic Reconstruction

Four nuclear (myh6, Ptr, ENC1, tbr) and two mitochondrial (nd4, cyt *b*) markers were PCR amplified and sequenced for 14 notothenioid and 53 related acanthomorph fish species, and complemented with additional sequences from GenBank, Ensemble, and Genoscope to a total of 83 taxa (Text S2, Tables S4–S5). Phylogenetic analyses were conducted in GARLI-PART v0.97 [56] and RAxML v7.26 [57], and node support was assessed with nonparametric bootstraps. Detailed information on sample collection, marker selection, PCR amplification, and phylogenetic inferences are given in the Text S2, S3, S4, S5 and S6 and Fig. S1.

### Time Constraints used for Dating

A total of eight fossil, and two phylogeographic constraints were chosen to time-calibrate acanthomorph divergences. Following Benton & Donoghue [58], we implemented time constraints for the origin of Tetraodontidae (node J; *Takifugu*-*Tetraodon* divergence; 56.0−32.25 Ma) and for the split between Gasterosteiformes and Tetraodontiformes (node G; 150.9−96.9 Ma). The minimum age for the Gasterosteiformes-Tetraodontiformes divergence is derived from the oldest known member of the tetraodontiform lineage, *Plectocretacicus clarae*, which also represents the oldest known percomorph. Also, the maximum constraint for divergence of gasterosteiform and tetraodontiform lineages is provided by the earliest euteleost record, *e.g. Tischlingerichthys viohli*. Therefore, we applied the same lower and upper bounds for the divergence of all Percomorpha (not including *Dicrolene introniger*, as the phylogenetic position of Ophidiiformes remains unclear [26]; node C). Uniform priors between minimum and maximum age of divergence were used for these constraints. In addition, we constrained five family divergences, which we expected to have relatively early fossil records in at least one of the descending lineages (Fig. S3). Thus, our analysis accounted for polymixiid fossils from the Cenomanian (node A; ≥93.5 Ma), a zeid fossil from the Thanetian (node B; ≥55.8 Ma), gempylid and scombrid fossils from the Danian (node D; ≥61.7 Ma), a chaunacid fossil from the Bartonian (node H; ≥37.2 Ma), and a monacanthid fossil from the Ypresian (node I; ≥48.6 Ma). All used fossils are referenced in detail in Text S7. Lognormal priors were assigned to the above fossil constraints with hard lower bounds reflecting the age of the respective fossil, and soft upper bounds (Table S5, Fig. 2). Phylogeographic constraints for cichlid and labrid divergences were derived from the breakup of Gondwana, and from the closure of the connection between the Mediterranean and the Indian Ocean. We added the separation of Africa and South America as effective time constraint for the split between African and neotropical cichlids (node E), assuming vicariant divergence. Seafloor spreading in the South Atlantic started as early as 133 Myr ago [59] and a continuous North/South Atlantic Ocean presumably existed ∼100 Myr ago [60], hence, we applied a normally distributed prior between 121.8 and 98.2 Ma (95% cumulative probability; mean: 110.0 Ma) to constrain cichlid divergence. The split between *Labrus* and both *Ctenolabrus* and *Tautogolabrus* (node F) represents the diversification of the labrid tribe Labrini [27]. Based on molecular clock calibrations and fossil evidence (Text S7) showing that Labrini were present in the Mediterranean 14.0 Ma, it has been suggested that the ancestor of Labrini migrated from the Indopacific into the Mediterranean prior to the closure of this seaway, 20.5−19.5 Ma [27]. Therefore, we constrained the diversification of Labrini with a uniform prior between 20.5 and 14.0 Ma.

### Dating of Acanthomorph Divergences

In order to date notothenioid and non-notothenioid acanthomorph divergences, we generated time-calibrated phylogenies with BEAST v1.5.3 [61]. All BEAST runs were performed using mitochondrial and nuclear sequence alignments as separate partitions with unlinked substitution models. We employed a relaxed molecular clock model with branch rates drawn independently from a lognormal distribution [62], ten time constraints (Table S5), and the reconstructed birth-death process [63] as a tree prior (see Fig. S4 for substitution rates). The applicability of relaxed molecular clocks for cold-adapted organisms is discussed in Text S8. After optimization of operators according to preliminary run results, three different substitution models were implemented and evaluated. We included the codon position-based HKY_112_+CP_112_+Γ_112_ model [64], in which all parameters are estimated independently for the first two and for the third codon positions. We also added the GTR_112_+CP_112_+Γ_112_ model, using the same partitions. In a third set, we implemented HKY+I+Γ for the first two mitochondrial codon positions, TVM+Γ for the third mitochondrial codon position, K80+I+Γ for the first nuclear codon position, and GTR+Γ for the third nuclear codon position, as selected by BIC. For each of the three setting, we performed 20 independent analyses of 20 million generations each, discarding the first 2 million generations of every replicate as burnin. Replicate results were combined in LogCombiner v1.5.3 after removing the burnin. Convergence of run replicates was confirmed by effective sample sizes (ESS) >1200 for all parameters and by visual inspection of traces within and between replicates in Tracer v1.5. Substitution models were evaluated on the basis of Bayes Factors, again, as implemented in Tracer [65]. Bayes Factors provided ‘very strong’ [66] evidence that the substitution model combination selected by BIC was better-fitting than both the HKY_112_+CP_112_+Γ_112_ (log 10 BF 350.6) and GTR_112_+CP_112_+Γ_112_ (log 10 BF 276.0) models, and thus the BIC combination was used for all subsequent analyses. In order to assess the reliability of every individual time constraint, we conducted a cross-validation, whereby we relaxed constraints one by one, and estimated divergence dates of relaxed constraints based on all other constraints (Fig. 2). BEAST runs were conducted as described above, but using 5 run replicates per cross-validation. We found good fit of posterior and prior distributions for constraints B, G, H, I, and J. Subsequently, 20 run replicates were performed with identical settings, but excluding the five unreliable constraints A, C, D, E, and F (run ‘-ACDEF’ in Fig. 2). Posterior distributions of excluded constraints were again compared to their assumed prior distributions. After exclution of five constraints, node C (divergence of Percomorpha) provided adequate fit of posterior probability distribution to its suggested bounds [58], and was thus reincluded for yet another run with 20 independent replicates and unchanged settings (run ‘-ADEF’ in Fig. 2 and Tables S1–S2). ESS values for this run were >900 for all parameters. As for GARLI-PART and RAxML analyses, the last run was repeated after removal of *Serranus atricauda* from the dataset (‘-ADEF -*Serranus atricauda*’ in Fig. 2 and Tables S1–S2), which had no impact on tree topology and little influence on node support (on average -0.19 BPP, Table S1) and divergence date estimates (average difference 0.57%, Table S2). All molecular data sets to date [22], [23], [31] failed to assign a reliable phylogenetic position to *G. gibberifrons* (node Y, BPP 0.64), thus indicating rapid divergence at the beginning of the notothenioid radiation. We also repeated the analysis without *G. gibberifrons* (8 replicates, unchanged settings) to obtain a minimum age estimate for the diversification of the Antarctic Clade that is robust to topological uncertainties. Maximum clade credibility trees were produced using TreeAnnotator v1.5.3.

## Supporting Information

Figure S1 Partitioned ML consensus tree of full mitochondrial genomes (A), and best ML phylogenies of single mitochondrial markers (B-D), estimated with RAxML. Sequence data were taken from [3], [4], [6]. Markers ND4 (B) and cyt *b* (C) phylogenies show better agreement with the full mitogenome topology than other markers of comparable sequence length (D).
(EPS)

Figure S2 Partitioned ML phylogeny of 83 acanthomorph fishes, based on the concatenated dataset of two mitochondrial (ND4, cyt *b*) and four nuclear genes (myh6, Ptr, ENC1, tbr). Tree topology and branch lengths are as estimated using GARLI-PART, and near-identical topologies were recovered with RAxML and BEAST analyses. Filled circles indicate > 98% BS support (as calculated with GARLI-PART) and > 0.99 BPP (according to BEAST run ‘-ADEF’), white circles represent nodes with BS support > 80% and >0.90 BPP. Split circles indicate different levels of BS (left half) and BPP (right half) support. All node support values are summarized in Table S3. Nodes that were used for fossil and phylogeographic constraints are labelled with letters A–J, basal percid and notothenioid nodes are labelled with letters T–Z.
(EPS)

Figure S3 Partitioned BI phylogeny, based on the concatenated data set, reduced to family level. Node heights correspond to mean age estimates. The time scale is divided into phanerozoic stages (grey shades), and the presence of skeletal (black bars) or otolith fossils (dark grey bars) in a stage is plotted on top of family branches. Unless otherwise noted, all fossil information is taken from [22]. Fossils with questionable taxonomic or stratigraphic assignments are indicated by dashed bars. Numbers in brackets indicate the number of species included in this study and the total number of species per family [31]. ^1^) Fossil used to constrain node D, however cross-validation results suggest unreliability of this constraint. As Gempylidae could be nested within Scombridae [25], fossils may have been misinterpreted. ^2^) [32], [33] ^3^) According to Santini & Tyler [34], the oldest tetraodontid is *Archaeotetraodon winterbottomi* from the Rupelian. *Eotetraodon pygmaeus*, previously assigned to Tetraodontidae [22], has been moved to the family Triodontidae ^4^) [35], [36].
(EPS)

Figure S4 Partitioned BI phylogeny of 83 acanthomorph fishes, based on the concatenated data set. Branch lengths are according to mean estimates of node ages (Table S4). Branch colors indicates substitution rates.
(EPS)

Table S1 Node support given as BS and BPP values for partitioned ML and BI phylogenetic reconstructions. Nodes marked with * were recovered in best ML tree topologies, but were not included in BS consensus trees. Nodes marked with. Nodes were labelled as in Fig. S2. BEAST analyses were based on six fossil constraints (run ‘-ADEF’). Exclusion of *Serranus atricauda* from the data set had little effect on node support.
(DOC)

Table S2 Divergence date estimates, estimated in BEAST on the basis of six reliable fossil calibrations (run ‘-ADEF’). For this analysis, time constraints were applied to nodes marked with *. Labels refer to nodes in Fig. S2. Exclusion of *Serranus atricauda* from the data set had negligible effects on age estimates. All dates are given in Ma.
(DOC)

Table S3 Estimates for the onset of the radiation of the AFGP-bearing Antarctic Clade (node X) when individual node constraints were removed during the constraint cross-validation. All dates are given in Ma.
(DOC)

Table S4 Genbank accession numbers for all sequences used for phylogenetic analyses. Sequences HM049934-HM050270 were produced as part of this study. * Nuclear *T. rubripes* and *T. nigroviridis* sequences were extracted from Ensembl (www.ensembl.org) and Genoscope (www.genoscope.cns.fr) genome browsers (Table S2).
(DOC)

Table S5 Ensembl and Genoscope identifiers of *Takifugu rubripes* and *Tetraodon nigroviridis* sequences. *T. rubripes* Ensembl identifiers were taken from [5], while *T. nigroviridis* Genoscope identifiers and sequences were found by BLAT-search against the *T. nigroviridis* genome, using the entire *T. rubripes* sequences as search templates.
(DOC)

Text S1 (DOC)

Text S2 (DOC)

Text S3 (DOC)

Text S4 (DOC)

Text S5 (DOC)

Text S6 (DOC)

Text S7 (DOC)

Text S8 (DOC)

## References

[1] Simpson GG (1953). The major features of evolution..

[2] Schluter D (2000). The ecology of adaptive radiation..

[3] Yoder JB, Clancey E, Des Roches S, Eastman JM, Gentry L (2010). Ecological opportunity and the origin of adaptive
radiations.. J Evol Biol.

[4] Heard SB, Hauser DL (1995). Key evolutionary innovations and their ecological
mechanisms.. Hist Biol.

[5] Lack D (1947). Darwin’s finches..

[6] Salzburger W, Mack T, Verheyen E, Meyer A (2005). Out of Tanganyika: Genesis, explosive speciation, key-innovations
and phylogeography of the haplochromine cichlid fishes.. BMC Evol Biol.

[7] Salzburger W (2009). The interaction of sexually and naturally selected traits in the
adaptive radiations of cichlid fishes.. Molecular Ecology.

[8] Seehausen O (2006). African cichlid fish: a model system in adaptive radiation
research.. Proc R Soc B.

[9] Alfaro ME, Brock CD, Banbury BL, Wainwright PC (2009). Does evolutionary innovation in pharyngeal jaws lead to rapid
lineage diversification in labrid fishes?. BMC Evol Biol.

[10] Losos JB (2009). Lizards in an evolutionary tree: ecology and adaptive radiation
of anoles..

[11] Eastman JT (2005). The nature of the diversity of Antarctic fishes.. Polar Biol.

[12] Chen L, DeVries AL, Cheng C-HC (1997). Evolution of antifreeze glycoprotein gene from a trypsinogen gene
in Antarctic notothenioid fish.. Proc Natl Acad Sci U S A.

[13] Pointer MA, Cheng CH, Bowmaker JK, Parry JW, Soto N (2005). Adaptations to an extreme environment: retinal organisation and
spectral properties of photoreceptors in Antarctic notothenioid
fish.. J Exp Biol.

[14] Hofmann GE, Lund SG, Place SP, Whitmer AC (2005). Some like it hot, some like it cold: the heat shock response is
found in New Zealand but not Antarctic notothenioid fishes.. J Exp Mar Biol Ecol.

[15] Eastman JT (1993). Antarctic fish biology: evolution in a unique
environment..

[16] Bilyk KT, DeVries AL (2010). Freezing avoidance of the Antarctic icefishes (Channichthyidae)
across thermal gradients in the Southern Ocean.. Polar Biol.

[17] Cheng C-HC, Chen L, Near TJ, Jin Y (2003). Functional antifreeze glycoprotein genes in temperate-water New
Zealand nototheniid fish infer an Antarctic evolutionary
origin.. Mol Biol Evol.

[18] Pagani M, Zachos JC, Freeman KH, Tipple B, Bohaty S (2005). Marked decline in atmospheric carbon dioxide concentrations
during the Paleogene.. Science.

[19] Zachos JC, Dickens GR, Zeebe RE (2008). An early Cenozoic perspective on greenhouse warming and
carbon-cycle dynamics.. Nature,.

[20] Cape Roberts Science Team (2000). Studies from the Cape Roberts Project, Ross Sea, Antarctica.
Initial report on CRP-3.. Terra Antarctica,.

[21] Olney MP, Bohaty SM, Harwood DM, Scherer RP (2009). *Creania lacyae* gen. nov. et sp. nov. and
*Synedropsis cheethamii* sp. nov., fossil indicators of
Antarctic sea ice?. Diatom Res.

[22] Near TJ (2004). Estimating divergence times of notothenioid fishes using a
fossil-calibrated molecular clock.. Antarct Sci.

[23] Bargelloni L, Marcato S, Zane L, Patarnello T (2004). Mitochondrial phylogeny of notothenioids: a molecular approach to
Antarctic fish evolution and biogeography.. Syst Biol.

[24] Chen W-J, Bonillo C, Lecointre G, di Prisco G, Pisano E, Clarke A (1998). Phylogeny of the Channichthyidae (Notothenioidei, Teleostei)
based on two mitochondrial genes.. Fishes of Antarctica.

[25] Bargelloni L, Ritchie PA, Paternello T, Battaglia B, Lambert DM (1994). Molecular evolution at subzero temperatures: mitochondrial and
nuclear phylogenies of fishes from Antarctica (suborder Notothenioidei), and
the evolution of antifreeze glycopeptides.. Mol Biol Evol.

[26] Miya M, Takeshima H, Endo H, Ishiguro NB, Inoue JG (2003). Major patterns of higher teleostean phylogenies: a new
perspective based on 100 complete mitochondrial DNA
sequences.. Mol Phylogenet Evol.

[27] Hanel R, Westneat MW, Sturmbauer C (2002). Phylogenetic relationships, evolution of broodcare behavior, and
geographic speciation in the wrasse tribe Labrini.. J Mol Evol.

[28] Kazancioglu E, Near TJ, Hanel R, Wainwright PC (2009). Influence of feeding functional morphology and sexual selection
on diversification rate in parrotfishes (Scaridae).. Proc R Soc B.

[29] Smith WL, Craig MT (2007). Casting the percomorph net widely: the importance of broad
taxonomic sampling in the search for the placement of serranid and percid
fishes.. Copeia.

[30] Dettaï A, Lecointre G (2005). Further support for the clades obtained by multiple molecular
phylogenies in the acanthomorph bush.. CR Biol.

[31] Near TJ, Cheng C-HC (2008). Phylogenetics of notothenioid fishes (Teleostei: Acanthomorpha):
Inferences from mitochondrial and nuclear gene sequences.. Mol Phylogenet Evol.

[32] Near TJ, Bolnick DI, Wainwright PC (2005). Fossil calibrations and molecular divergence time estimates in
centrarchid fishes (Teleostei: Centrarchidae).. Evolution.

[33] Patterson C, Benton MJ (1993). Osteichthyes: Teleostei.. The fossil record 2.

[34] Eastman JT, Grande L (1991). Late Eocene gadiform (Teleostei) skull from Seymour Island,
Antarctic Peninsula.. Antarct Sci.

[35] Balushkin AV (1994). Fossil notothenioid, and not gadiform, fish *Proeleginops
grandeastmanorum* gen. nov. sp. nov. (Perciformes,
Notothenioidei, Eleginopidae) from the late Eocene found in Seymour Island
(Antarctica).. J Ichthyol.

[36] Shevenell AE, Kennett JP, Lea DW (2004). Middle Miocene Southern Ocean cooling and Antarctic cryosphere
expansion.. Science.

[37] Shevenell AE, Kennett JP (2007). Cenozoic Antarctic cryosphere evolution: tales from deep-sea
sedimentary records.. Deep-Sea Res II.

[38] DeConto RM, Pollard D (2003). Rapid Cenozoic glaciation of Antarctica induced by declining
atmospheric CO_2_.. Nature.

[39] Zachos JC, Quinn TM, Salamy KA (1996). High resolution (10^4^ years) deep-sea foraminiferal
stable isotope records of the Eocene-Oligocene climate
transition.. Paleoceanography.

[40] DeConto RM, Pollard D, Harwood D (2007). Sea ice feedback and Cenozoic evolution of Antarctic climate and
ice sheets.. Paleoceanography.

[41] Barker PF, Filippelli GM, Florindo F, Martin EE, Scher HD (2007). Onset and role of the Antarctic Circumpolar
Current.. Deep-Sea Res II.

[42] Nong GT, Najjar RG, Seidov D, Peterson WH (2000). Simulation of ocean temperature change due to the opening of
Drake Passage.. Geophys Res Lett.

[43] Naish TR, Woolfe KJ, Barret PJ, Wilson GC, Bohaty SM (2001). Orbitally induced oscillations in the East Antarctic ice sheet at
the Oligocene/Miocene boundary.. Nature.

[44] Cheng C-HC, di Prisco G, Pisano E, Clarke A (1998). Origin and mechanism of evolution of antifreeze glycoproteins in
polar fishes.. Fishes of Antarctica. A biological overview.

[45] Chen L, DeVries AL, Cheng C-HC (1997). Convergent evolution of antifreeze glycoproteins in Antarctic
notothenioid fish and Arctic cod.. Proc Natl Acad Sci U S A.

[46] O’Grady SM, Schrag JD, Raymond JA, DeVries AL (1982). Comparison of antifreeze glycopeptides from arctic and antarctic
fishes.. J Exp Zool.

[47] Fletcher GL, Hew CL, Davies PL (2001). Antifreeze proteins of teleost fishes.. Annu Rev Physiol.

[48] Bakke I, Johansen SD (2005). Molecular phylogenetics of Gadidae and related Gadiformes based
on mitochondrial DNA sequences.. Mar Biotechnol.

[49] Anderson ME (1994). Systematics and osteology of the Zoarcidae (Teleostei:
Perciformes).. Ichthyol Bull JLB Smith Inst Ichthyol.

[50] Graham LA, Lougheed SC, Ewart KV, Davies PL (2008). Lateral transfer of a Lectin-like antifreeze protein gene in
fishes.. PLoS ONE.

[51] Salzburger W (2008). To be or not to be a hamlet pair in sympatry.. Mol Ecol.

[52] Balushkin AV (2000). Morphology, classification, and evolution of notothenioid fishes
of the Southern Ocean (Notothenioidei, Perciformes).. J Ichthyol.

[53] Anderson JB (1999). Antarctic Marine Geology Cambridge: Cambridge University Press..

[54] Kennett JP, Exon NF, Exon NF, Kennett JP, Malone MJ (2004). Paleoceanographic evolution of the Tasmanian Seaway and its
climatic implications.. The Cenozoic Southern Ocean: Tectonics, sedimentation, and climate
change between Australia and Antarctica. Geophysical Monograph.

[55] Stickley CE, Brinkhuis H, Schellenberg SA, Sluijs A, Roehl U (2004). Timing and nature of the deepening of the Tasmanian
Gateway.. Paleoceanography.

[56] Zwickl DJ (2006). Genetic algorithm approaches for the phylogenetic analysis of
large biological sequence datasets under the maximum likelihood
criterion..

[57] Stamatakis A (2006). RAxML-VI-HPC: maximum likelihood-based phylogenetic analyses with
thousands of taxa and mixed models.. Bioinformatics.

[58] Benton MJ, Donoghue P (2007). Paleontological evidence to date the tree of
life.. Mol Biol Evol.

[59] Storey BC (1995). The role of mantle plumes in continental breakup: case histories
from Gondwanaland.. Nature.

[60] Sereno PC, Wilson JA, Conrad JL (2004). New dinosaurs link southern landmasses in the
Mid-Cretaceous.. Proc R Soc B.

[61] Drummond AJ, Rambaut A (2007). BEAST: Bayesian evolutionary analysis by sampling
trees.. BMC Evol Biol.

[62] Drummond AJ, Ho SYW, Philips MJ, Rambaut A (2006). Relaxed phylogenetics and dating with confidence.. PLoS Biol.

[63] Gernhard T (2008). The conditioned reconstructed process.. J Theor Biol.

[64] Shapiro B, Rambaut A, Drummond AJ (2006). Choosing appropriate substitution models for the phylogenetic
analysis of protein-coding sequences.. Mol Biol Evol.

[65] Suchard MA, Weiss RE, Sinsheimer JS (2001). Bayesian selection of continuous-time Markov Chain evolutionary
models.. Mol Biol Evol.

[66] Kass RE, Raftery AE (1995). Bayes Factors.. J Am Stat Assoc.

[67] Larter RD, Cunningham AP, Barker PF, Gohl K, Nitsche FO (2002). Tectonic evolution of the Pacific margin of Antarctica. 1. Late
Cretaceous tectonic reconstructions.. J Geophys Res.

[68] Livermore R, Nankivell A, Eagles G, Morris P (2005). Paleogene opening of Drake Passage.. Earth Planet Sc Lett.

[69] Henderiks J, Pagani M (2008). Coccolithophore cell size and the Paleogene decline in
atmospheric CO_2_.. Earth Planet Sc Lett.

