# Supplementary material for: On the Origin and Trigger of the Notothenioid Adaptive Radiation
Source: PLoS One. 2011 Apr 18;6(4):e18911. doi: 10.1371/journal.pone.0018911 (PMC3078932; doi:10.1371/journal.pone.0018911)
Supplement: Table S1 — Node support given as BS and BPP values for partitioned ML and BI phylogenetic reconstructions. Nodes marked with * were recovered in best ML tree topologies, but were not included in BS consensus trees. Nodes marked with. Nodes were labelled as in Fig. S2. BEAST analyses were based on six fossil constraints (run ‘-ADEF’). Exclusion of Serranus atricauda from the data set had little effect on node support. (DOC) [file pone.0018911.s005.doc]

| Node | full taxa set | | | without *Serranus atricauda* | | |
| --- | --- | --- | --- | --- | --- | --- |
|  | GARLI-PART | RAxML | BEAST | GARLI-PART | RAxML | BEAST |
| A | 62 | 38 | 0.94 | 56 | 38 | 0.95 |
| B | 93 | 86 | 1 | 86 | 85 | 1 |
| C | 100 | 100 | 0.92 | 100 | 100 | 91 |
| D | 100 | 100 | 1 | 100 | 100 | 1 |
| E | 100 | 100 | 1 | 100 | 100 | 1 |
| F | 100 | 100 | 1 | 100 | 100 | 1 |
| G | 75 | 79 | 0.98 | 77 | 67 | 0.98 |
| H | 100 | 100 | 1 | 100 | 100 | 1 |
| I | 100 | 100 | 1 | 100 | 100 | 1 |
| J | 100 | 100 | 1 | 100 | 100 | 1 |
| T | 100 | 100 | 1 | 100 | 100 | 1 |
| U | 100 | 100 | 1 | 100 | 100 | 1 |
| V | 86 | 83 | 1 | 84 | 83 | 1 |
| W | 100 | 100 | 1 | 100 | 100 | 1 |
| X | 100 | 100 | 1 | 100 | 100 | 1 |
| Y | 55 | 53 | 0.64 | 50 | 57 | 0.65 |
| Z | 98 | 97 | 1 | 97 | 98 | 1 |
| 1 | 100 | 100 | 0.99 | 100 | 100 | 0.99 |
| 2 | 100 | 100 | 1 | 100 | 100 | 1 |
| 3 | 100 | 100 | 0.45 | 100 | 100 | 0.47 |
| 4 | 50 | 61 | 0.84 | 49 | 63 | 0.86 |
| 5 | 57 | 78 | 0.89 | 64 | 74 | 0.89 |
| 6 | 92 | 94 | 1 | 91 | 94 | 1 |
| 7 | 97 | 99 | 0.99 | 98 | 99 | 0.99 |
| 8 | 100 | 100 | 1 | 100 | 100 | 1 |
| 9 | * | 21 | 0.60 | * | 16 | 0.60 |
| 10 | 48 | 47 | 0.89 | 57 | 45 | 0.85 |
| 11 | 65 | 90 | 1 | 75 | 89 | 1 |
| 12 | 85 | 94 | 1 | 90 | 94 | 1 |
| 13 | 100 | 100 | 1 | 100 | 100 | 1 |
| 14 | 100 | 100 | 1 | 100 | 100 | 1 |
| 15 | 100 | 100 | 1 | 100 | 100 | 1 |
| 16 | 94 | 98 | 1 | 99 | 98 | 1 |
| 17 | 100 | 100 | 1 | 100 | 100 | 1 |
| 18 | 92 | 98 | 1 | 95 | 96 | 1 |
| 19 | 100 | 100 | 1 | 100 | 100 | 1 |
| 20 | 100 | 100 | 1 | 100 | 100 | 1 |
| 21 | 92 | 96 | 1 | 90 | 94 | 1 |
| 22 | 100 | 100 | 1 | 100 | 100 | 1 |
| 23 | 95 | 99 | 1 | 97 | 97 | 1 |
| 24 | 72 | 82 | 1 | 71 | 83 | 1 |
| 25 | * | 56 | 0.69 | * | 61 | 0.61 |
| 26 | 79 | 85 | 1 | 79 | 84 | 1 |
| 27 | 33 | - | 0.64 | - | - | 0.59 |
| 28 | 84 | 86 | 1 | 81 | 92 | 1 |
| 29 | 80 | 91 | 1 | 87 | 96 | 1 |
| 30 | 92 | 94 | 1 | 95 | 94 | 1 |
| 31 | 42 | 46 | 0.98 | 60 | 46 | 0.98 |
| 32 | 100 | 100 | 1 | 100 | 100 | 1 |
| 33 | 100 | 100 | 1 | 100 | 100 | 1 |
| 34 | 51 | 48 | 0.99 | 66 | 54 | 1 |
| 35 | 62 | 71 | 0.98 | 55 | 54 | 0.98 |
| 36 | 51 | 55 | 0.68 | 55 | 56 | 0.72 |
| 37 | 100 | 100 | 1 | 100 | 100 | 1 |
| 38 | 100 | 100 | 1 | 100 | 100 | 1 |
| 39 | 100 | 98 | 1 | 100 | 97 | 1 |
| 40 | 57 | 62 | 0.84 | 54 | 62 | 0.86 |
| 41 | 100 | 100 | 1 | 100 | 100 | 1 |
| 42 | 85 | 85 | 1 | 81 | 85 | 1 |
| 43 | 80 | 85 | 1 | 77 | 84 | 1 |
| 44 | 100 | 98 | 1 | 100 | 99 | 1 |
| 45 | 59 | 62 | 0.88 | 65 | 65 | 0.91 |
| 46 | 100 | 100 | 1 | 100 | 100 | 1 |
| 47 | 19 | 26 | 0.83 | 27 | 30 | 0.76 |
| 48 | 71 | 56 | 1 | 72 | 70 | 0.89 |
| 49 | 35 | 32 | 0.78 | - | - | - |
| 50 | 100 | 100 | 1 | 100 | 100 | 1 |
| 51 | 100 | 100 | 1 | 100 | 100 | 1 |
| 52 | 100 | 100 | 1 | 100 | 100 | 1 |
| 53 | 100 | 100 | 1 | 100 | 100 | 1 |
| 54 | 94 | 97 | 1 | 94 | 97 | 1 |
| 55 | 76 | 91 | 1 | 85 | 94 | 1 |
| 56 | 100 | 100 | 1 | 100 | 100 | 1 |
| 57 | 100 | 100 | 1 | 100 | 100 | 1 |
| 58 | 100 | 100 | 1 | 100 | 100 | 1 |
| 59 | 92 | 89 | 1 | 87 | 89 | 1 |
| 60 | 63 | 84 | 0.95 | 59 | 87 | 0.96 |
| 61 | 90 | 95 | 1 | 96 | 95 | 1 |
| 62 | 98 | 98 | 1 | 96 | 98 | 1 |
| 63 | 100 | 100 | 1 | 100 | 100 | 1 |
| 64 | 100 | 100 | 1 | 100 | 100 | 1 |
