# Supplementary material for: On the Origin and Trigger of the Notothenioid Adaptive Radiation
Source: PLoS One. 2011 Apr 18;6(4):e18911. doi: 10.1371/journal.pone.0018911 (PMC3078932; doi:10.1371/journal.pone.0018911)
Supplement: Table S2 — Divergence date estimates, estimated in BEAST on the basis of six reliable fossil calibrations (run ‘-ADEF’). For this analysis, time constraints were applied to nodes marked with *. Labels refer to nodes in Fig. S2. Exclusion of Serranus atricauda from the data set had negligible effects on age estimates. All dates are given in Ma. (DOC) [file pone.0018911.s006.doc]

| Node | -ADEF | | | -ADEF -*Serranus atricauda* | | |
| --- | --- | --- | --- | --- | --- | --- |
|  | 95% HPD upper | Mean | 95% HPD lower | 95% HPD upper | Mean | 95% HPD lower |
| A | 133.05 | 99.16 | 70.51 | 130.78 | 98.12 | 69.53 |
| B* | 95.52 | 72.42 | 56.57 | 93.76 | 71.82 | 56.74 |
| C* | 150.89 | 131.18 | 114.66 | 150.89 | 131.20 | 114.62 |
| D | 79.15 | 46.14 | 18.36 | 82.56 | 47.34 | 18.64 |
| E | 80.71 | 59.70 | 40.24 | 81.24 | 59.36 | 39.26 |
| F | 52.40 | 34.89 | 19.31 | 52.12 | 34.79 | 19.04 |
| G* | 124.98 | 110.13 | 96.90 | 125.44 | 110.40 | 96.90 |
| H* | 56.32 | 44.89 | 37.54 | 56.36 | 44.86 | 37.54 |
| I* | 66.31 | 56.44 | 49.19 | 66.36 | 56.48 | 49.19 |
| J* | 53.53 | 42.96 | 32.26 | 53.53 | 42.98 | 32.25 |
| T | 54.71 | 41.15 | 28.54 | 55.08 | 41.36 | 28.83 |
| U | 89.44 | 71.44 | 54.41 | 89.45 | 71.72 | 54.41 |
| V | 80.26 | 63.01 | 46.70 | 80.21 | 63.09 | 46.52 |
| W | 56.86 | 42.93 | 29.81 | 57.17 | 43.09 | 29.99 |
| X | 31.94 | 23.88 | 16.72 | 31.84 | 24.06 | 16.80 |
| Y | 28.20 | 21.36 | 15.26 | 28.21 | 21.52 | 15.38 |
| Z | 20.02 | 14.71 | 9.93 | 20.07 | 14.86 | 10.04 |
| root | 194.47 | 158.13 | 124.65 | 193.97 | 157.61 | 124.51 |
| 1 | 151.51 | 113.60 | 79.27 | 150.85 | 112.86 | 78.96 |
| 2 | 20.77 | 11.90 | 4.54 | 20.62 | 11.93 | 4.78 |
| 3 | 170.20 | 134.51 | 97.80 | 168.79 | 133.79 | 96.93 |
| 4 | 143.15 | 125.02 | 107.81 | 143.31 | 124.97 | 107.84 |
| 5 | 139.52 | 120.30 | 102.26 | 139.41 | 120.09 | 102.18 |
| 6 | 124.30 | 95.53 | 64.82 | 124.39 | 95.59 | 64.83 |
| 7 | 128.41 | 107.94 | 88.07 | 129.05 | 107.83 | 88.05 |
| 8 | 47.99 | 29.03 | 12.29 | 49.42 | 29.41 | 12.67 |
| 9 | 124.33 | 103.79 | 83.66 | 125.24 | 103.63 | 83.65 |
| 10 | 116.87 | 95.95 | 75.88 | 117.13 | 95.64 | 75.04 |
| 11 | 102.38 | 77.94 | 54.02 | 102.31 | 77.30 | 52.52 |
| 12 | 96.51 | 75.92 | 55.83 | 96.80 | 75.71 | 55.95 |
| 13 | 57.36 | 39.22 | 22.39 | 57.23 | 39.33 | 22.25 |
| 14 | 45.29 | 28.36 | 13.12 | 44.49 | 28.10 | 12.95 |
| 15 | 47.18 | 32.45 | 19.47 | 46.58 | 32.25 | 19.13 |
| 16 | 29.43 | 19.44 | 10.51 | 29.23 | 19.32 | 10.51 |
| 17 | 15.90 | 9.65 | 4.21 | 16.02 | 9.64 | 4.26 |
| 18 | 131.03 | 114.62 | 99.28 | 131.34 | 114.80 | 99.30 |
| 19 | 104.13 | 82.56 | 61.47 | 103.96 | 82.38 | 60.26 |
| 20 | 68.95 | 46.61 | 25.72 | 68.01 | 46.27 | 25.26 |
| 21 | 84.66 | 63.05 | 42.06 | 84.97 | 63.03 | 41.70 |
| 22 | 19.80 | 11.77 | 4.73 | 19.75 | 11.66 | 4.88 |
| 23 | 115.64 | 99.59 | 85.17 | 116.31 | 99.71 | 85.26 |
| 24 | 105.84 | 85.20 | 62.96 | 106.78 | 85.55 | 63.98 |
| 25 | 96.57 | 74.13 | 49.94 | 97.46 | 74.45 | 50.17 |
| 26 | 110.04 | 94.14 | 80.43 | 109.91 | 94.21 | 79.87 |
| 27 | 105.80 | 89.46 | 74.66 | 106.04 | 89.57 | 74.57 |
| 28 | 93.42 | 75.80 | 57.98 | 93.44 | 75.91 | 57.92 |
| 29 | 97.90 | 82.92 | 69.41 | 97.64 | 82.84 | 69.04 |
| 30 | 119.69 | 102.91 | 88.28 | 120.01 | 102.84 | 88.15 |
| 31 | 110.83 | 90.16 | 69.12 | 111.64 | 90.26 | 69.35 |
| 32 | 74.98 | 51.24 | 29.00 | 74.65 | 51.33 | 29.39 |
| 33 | 41.51 | 25.22 | 11.09 | 42.11 | 25.44 | 11.24 |
| 34 | 110.90 | 94.68 | 79.48 | 111.17 | 94.43 | 79.34 |
| 35 | 106.47 | 90.10 | 74.97 | 106.57 | 89.84 | 74.74 |
| 36 | 102.68 | 86.48 | 71.55 | 102.64 | 86.30 | 71.30 |
| 37 | 26.35 | 16.21 | 7.71 | 26.59 | 16.32 | 7.79 |
| 38 | 6.66 | 3.83 | 1.55 | 6.68 | 3.89 | 1.50 |
| 39 | 90.32 | 74.58 | 59.89 | 90.14 | 74.44 | 59.82 |
| 40 | 84.84 | 69.47 | 54.87 | 84.73 | 69.28 | 54.89 |
| 41 | 65.91 | 52.29 | 39.60 | 65.43 | 52.13 | 39.64 |
| 42 | 56.82 | 44.37 | 32.54 | 56.48 | 44.17 | 32.61 |
| 43 | 49.42 | 36.90 | 24.57 | 49.55 | 36.79 | 24.81 |
| 44 | 43.38 | 31.93 | 21.39 | 43.25 | 31.84 | 20.98 |
| 45 | 37.72 | 26.13 | 15.43 | 37.24 | 25.94 | 15.12 |
| 46 | 48.77 | 30.37 | 13.78 | 48.95 | 30.21 | 13.70 |
| 47 | 108.03 | 91.72 | 76.49 | 108.54 | 91.90 | 76.68 |
| 48 | 99.51 | 81.45 | 63.96 | 100.36 | 81.79 | 64.05 |
| 49 | 93.26 | 74.02 | 55.05 | - | - | - |
| 50 | 26.12 | 15.24 | 5.89 | 25.94 | 15.20 | 6.17 |
| 51 | 41.90 | 29.67 | 18.36 | 42.37 | 29.94 | 18.75 |
| 52 | 28.56 | 18.41 | 9.17 | 28.66 | 18.61 | 9.56 |
| 53 | 1.42 | 0.77 | 0.25 | 1.45 | 0.79 | 0.25 |
| 54 | 45.16 | 33.00 | 21.77 | 45.36 | 33.09 | 21.83 |
| 55 | 36.14 | 25.56 | 15.82 | 36.19 | 25.49 | 15.59 |
| 56 | 26.24 | 16.87 | 8.51 | 26.03 | 16.73 | 8.16 |
| 57 | 9.34 | 5.38 | 2.13 | 9.37 | 5.42 | 2.18 |
| 58 | 15.16 | 10.33 | 6.06 | 15.28 | 10.49 | 6.13 |
| 59 | 11.02 | 6.93 | 3.37 | 11.03 | 7.00 | 3.37 |
| 60 | 25.08 | 18.74 | 12.94 | 25.16 | 18.89 | 13.13 |
| 61 | 17.34 | 11.78 | 6.65 | 17.34 | 11.91 | 6.63 |
| 62 | 17.10 | 12.28 | 7.77 | 17.24 | 12.44 | 7.91 |
| 63 | 9.35 | 6.20 | 3.35 | 9.43 | 6.32 | 3.45 |
| 64 | 3.96 | 2.36 | 0.97 | 4.13 | 2.43 | 1.00 |
