# Supplementary material for: On the Origin and Trigger of the Notothenioid Adaptive Radiation
Source: PLoS One. 2011 Apr 18;6(4):e18911. doi: 10.1371/journal.pone.0018911 (PMC3078932; doi:10.1371/journal.pone.0018911)
Supplement: Table S3 — Estimates for the onset of the radiation of the AFGP-bearing Antarctic Clade (node X) when individual node constraints were removed during the constraint cross-validation. All dates are given in Ma. (DOC) [file pone.0018911.s007.doc]

| Constraint Set | 95% HPD upper | Mean | 95% HPD lower |
| --- | --- | --- | --- |
| Full set | 35.4 | 26.7 | 18.7 |
| -A | 34.3 | 25.9 | 17.8 |
| -B | 35.4 | 26.8 | 19.0 |
| -C | 39.0 | 29.3 | 20.0 |
| -D | 34.8 | 26.3 | 18.1 |
| -E | 31.3 | 23.4 | 16.4 |
| -F | 36.2 | 27.6 | 19.8 |
| -G | 34.6 | 26.2 | 18.5 |
| -H | 35.5 | 26.8 | 18.8 |
| -I | 36.0 | 27.0 | 19.0 |
| -J | 35.4 | 26.8 | 18.8 |
| -ACDEF | 34.2 | 25.0 | 16.8 |
| -ADEF | 31.9 | 23.9 | 16.7 |
