# Supplementary material for: On the Origin and Trigger of the Notothenioid Adaptive Radiation
Source: PLoS One. 2011 Apr 18;6(4):e18911. doi: 10.1371/journal.pone.0018911 (PMC3078932; doi:10.1371/journal.pone.0018911)
Supplement: Table S4 — Genbank accession numbers for all sequences used for phylogenetic analyses. Sequences HM049934-HM050270 were produced as part of this study. * Nuclear T. rubripes and T. nigroviridis sequences were extracted from Ensembl (www.ensembl.org) and Genoscope (www.genoscope.cns.fr) genome browsers (Table S2). (DOC) [file pone.0018911.s008.doc]

| Taxa | ND4 | CytB | myh6 | Ptr | ENC1 | tbr1 |
| --- | --- | --- | --- | --- | --- | --- |
| *Amphilophus citrinellus* | HM050088 | AB018985 | HM050030 | HM050148 | HM049972 | HM050209 |
| *Andinoacara pulcher* | HM050087 | EF432944 | HM050029 | HM050147 | HM049971 | HM050208 |
| *Antigonia capros* | NC_003191 | NC_003191 | EF536307 | HM050149 | HM049973 | HM050210 |
| *Aphredoderus sayanus* | NC_004372 | NC_004372 | EU001908 | EU001962 | EU002019 | EU001990 |
| *Aulichthys japonicus* | NC_011569 | NC_011569 | AB445150 | AB445168 | AB445222 | AB445204 |
| *Aulorhynchus flavidus* | NC_010268 | NC_010268 | AB445151 | AB445169 | AB445223 | AB445205 |
| *Balistes capriscus* | HM050089 | EF392572 | HM050031 | HM050150 | HM049974 | HM050211 |
| *Bembrops greyi* | HM050090 | HM049934 | HM050032 | HM050151 | HM049975 | HM050212 |
| *Bembrops heterurus* | HM050091 | HM049935 | HM050033 | HM050152 | HM049976 | HM050213 |
| *Bovichtus diacanthus* | HM050092 | HM049936 | HM050034 | HM050153 | HM049977 | HM050214 |
| *Cephalopholis taeniops* | HM050093 | EF455991 | HM050035 | HM050154 | HM049978 | HM050215 |
| *Chaenocephalus aceratus* | HM050094 | HM049937 | HM050036 | HM050155 | HM050216 |  |
| *Champsocephalus gunnari* | HM050095 | HM049938 | HM050037 | HM050156 | HM049979 | HM050217 |
| *Chaunax suttkusi* | HM050096 | HM049939 | HM050038 | HM050157 | HM049980 | HM050218 |
| *Chionodraco hamatus* | HM050097 | HM049940 | HM050039 | HM050158 | HM049981 | HM050219 |
| *Coris julis* | HM050099 | HM049942 | HM050041 | HM050160 | HM049982 | HM050221 |
| *Coryphoblennius galerita* | HM050098 | HM049941 | HM050040 | HM050159 | HM050220 |  |
| *Cottus gobio* | HM050100 | AY116366 | HM050042 | HM050222 |  |  |
| *Ctenolabrus rupestris* | HM050101 | HM049943 | HM050043 | HM050161 | HM049983 | HM050223 |
| *Culaea inconstans* | NC_011577 | NC_011577 | AB445153 | AB445171 | AB445225 | AB445207 |
| *Dicrolene introniger* | HM050102 | HM049944 | HM050044 | HM050162 | HM049984 | HM050224 |
| *Eleginops maclovinus* | DQ526429 | DQ526429 | HM050045 | HM050163 | HM049985 | HM050225 |
| *Epinephelus aeneus* | HM050103 | DQ197950 | HM050046 | HM050164 | HM049986 | HM050226 |
| *Erythrocles monodi* | HM050104 | EF456004 | HM050047 | HM050165 | HM049987 | HM050227 |
| *Etheostoma caeruleum* | HM050105 | DQ465142 | HM050166 | HM049988 | HM050228 |  |
| *Etheostoma zonale* | HM050106 | AY964705 | HM050167 | HM049989 | HM050229 |  |
| *Euthynnus alletteratus* | NC_004530 | EF439531 | HM050048 | HM050168 | HM049990 | HM050230 |
| *Gadus morhua* | NC_002081 | EU877717 | EU001906 | EU001960 | EU002017 |  |
| *Gambusia affinis* | NC_004388 | NC_004388 | EU001907 | EU001961 | EU002018 | EU001989 |
| *Gasterosteus aculeatus* | AP002944 | AP002944 | AB445155 | AB445173 | AB445227 | AB445209 |
| *Gobionotothen gibberifrons* | HM050107 | HM049945 | HM050049 | HM050169 | HM049991 | HM050231 |
| *Gymnocephalus cernuus* | HM050108 | AF045356 | HM050050 | HM050170 | HM049992 | HM050232 |
| *Gymnocephalus schraetser* | HM050109 | HM049946 | HM050051 | HM050171 | HM049993 | HM050233 |
| *Harpagifer kerguelensis* | HM050110 | HM049947 | HM050052 | HM050172 | HM049994 | HM050234 |
| *Helicolenus dactylopterus* | HM050111 | EU492259 | HM050053 | HM050173 | HM049995 |  |
| *Hemichromis* sp. | HM050112 | HM049948 | HM050174 | HM049996 | HM050235 |  |
| *Hypoptychus dybowskii* | NC_004400 | NC_004400 | AB445149 | AB445167 | AB445221 | AB445203 |
| *Labrus merula* | HM050113 | HM049949 | HM050054 | HM050175 | HM050236 |  |
| *Lepidocybium flavobrunneum* | HM050114 | AM265576 | HM050055 | HM050176 | HM049997 | HM050237 |
| *Lepidonotothen larseni* | HM050115 | HM049950 | HM050056 | HM050177 | HM050238 |  |
| *Lepidonotothen squamifrons* | HM050116 | HM049951 | HM050057 | HM050178 | HM049998 | HM050239 |
| *Lophius vaillanti* | HM050117 | HM049952 | HM050058 | HM050179 | HM049999 | HM050240 |
| *Melanocetus johnsonii* | HM050118 | HM049953 | HM050059 | HM050180 | HM050241 |  |
| *Mugil cephalus* | NC_003182 | EU083840 | HM050060 | HM050000 | HM050242 |  |
| *Mugil curema* |  | EU715492 | EU001913 | EU001967 | EU002023 | EU001994 |
| *Mycteroperca fusca* | HM050119 | DQ197968 | HM050061 | HM050181 | HM050001 | HM050243 |
| *Neolamprologus modestus* | HM050120 | HM049954 | HM050062 | HM050182 | HM050002 | HM050244 |
| *Notothenia coriiceps* | HM050121 | HM049955 | HM050063 | HM050183 | HM050003 | HM050245 |
| *Opeatogenys gracilis* | HM050122 | HM049956 | HM050064 | HM050004 | HM050246 |  |
| *Oreochromis tanganicae* | HM050123 | HM049957 | HM050065 | HM050184 | HM050005 | HM050247 |
| *Oryzias latipes* | NC_004387 | AB084730 | EF032927 | EF032953 | EF032979 | EF032966 |
| *Pachycara crossacanthum* | HM050124 | HM049958 | HM050066 | HM050006 | HM050248 |  |
| *Pagrus auriga* | NC_005146 | DQ197974 | HM050067 | HM050185 | HM050007 | HM050249 |
| *Parachaenichthys charcoti* | HM050125 | HM049959 | HM050068 | HM050186 | HM050008 | HM050250 |
| *Paranthias colonus* | HM050126 | HM049960 | HM050187 | HM050009 |  |  |
| *Perca fluviatilis* | HM050129 | AY929376 | HM050070 | HM050189 | HM050012 | HM050253 |
| *Percina caprodes* | HM050127 | DQ493490 |  | HM050010 | HM050251 |  |
| *Percina macrolepida* | NC_008111 | DQ493495 | HM050190 | HM050013 | HM050254 |  |
| *Peristedion cataphractum* | HM050128 | HM049961 | HM050069 | HM050188 | HM050011 | HM050252 |
| *Plectorhinchus mediterraneus* | HM050130 | DQ197979 | HM050071 | HM050191 | HM050014 | HM050255 |
| *Pleuronectes platessa* | HM050131 | EU224075 | EU001930 | HM050192 | EU002008 |  |
| *Pogonophryne scotti* | HM050132 | HM049962 | HM050072 | HM050193 | HM050256 |  |
| *Polymixia japonica* | NC_002648 | NC_002648 | EU001926 | EU001981 | EU002037 |  |
| *Polymixia nobilis* | HM050133 | DQ197980 | HM050073 | HM050194 | HM050015 | HM050257 |
| *Pseudaphritis urvillii* | HM050134 | HM049963 | HM050074 | HM050195 | HM050016 | HM050258 |
| *Salaria fluviatilis* | HM050135 | HM049964 | HM050075 | HM050196 | HM050017 | HM050259 |
| *Sander lucioperca* | HM050136 | HM049965 | HM050076 | HM050197 | HM050018 | HM050260 |
| *Sebastes marinus* | HM050137 | EF456022 | HM050077 | HM050019 | HM050261 |  |
| *Sebastes ruberrimus* | EU008930 | AF031501 | EU001929 | EU001984 | EU002040 | EU002007 |
| *Serranus atricauda* |  | EF439230 | HM050078 | HM050198 | HM050020 | HM050262 |
| *Sparisoma cretense* | HM050138 | HM049966 | HM050079 | HM050199 | HM050263 |  |
| *Spinachia spinachia* | NC_011582 | NC_011582 | AB445157 | AB445175 | AB445229 | AB445211 |
| *Stephanolepis hispidus* | HM050139 | HM049967 | HM050080 | HM050200 | HM050021 |  |
| *Takifugu rubripes* | NC_004299 | NC_004299 | * | * | * | * |
| *Tautogolabrus adspersus* | HM050140 | HM049968 | HM050081 | HM050201 | HM050022 | HM050264 |
| *Tetraodon nigroviridis* | NC_007176 | AP006046 | * | * | * | * |
| *Thalassoma pavo* | HM050141 | DQ198011 | HM050082 | HM050202 | HM050023 | HM050265 |
| *Trachurus picturatus* | HM050142 | EF392634 | HM050083 | HM050203 | HM050024 | HM050266 |
| *Trematomus newnesi* | HM050143 | HM049969 | HM050084 | HM050204 | HM050025 | HM050267 |
| *Tropheus moorii* | HM050144 | AB018990 | HM050205 | HM050026 | HM050268 |  |
| *Zeus faber* | NC_003190 | EU264027 | EU001927 | EU001982 | EU002038 |  |
| *Zingel streber* | HM050145 | HM049970 | HM050085 | HM050206 | HM050027 | HM050269 |
| *Zoarces viviparus* | HM050146 | EU492074 | HM050086 | HM050207 | HM050028 | HM050270 |
