# Supplementary material for: On the Origin and Trigger of the Notothenioid Adaptive Radiation
Source: PLoS One. 2011 Apr 18;6(4):e18911. doi: 10.1371/journal.pone.0018911 (PMC3078932; doi:10.1371/journal.pone.0018911)
Supplement: Table S5 — Ensembl and Genoscope identifiers of Takifugu rubripes and Tetraodon nigroviridis sequences. T. rubripes Ensembl identifiers were taken from [5], while T. nigroviridis Genoscope identifiers and sequences were found by BLAT-search against the T. nigroviridis genome, using the entire T. rubripes sequences as search templates. (DOC) [file pone.0018911.s009.doc]

| Taxa | myh6 | Ptr | ENC1 | tbr1 |
| --- | --- | --- | --- | --- |
| *T. rubripes* | SINFRUE00000644156 | SINFRUE00000786790 | SINFRUE00000681690 | SINFRUE00000673034 |
| *T. nigroviridis* | GSTENT00008412001 | GSTENT00035515001 | GSTENT00025143001 | GSTENT00030575001 |
