# Supplementary material for: On the Origin and Trigger of the Notothenioid Adaptive Radiation
Source: PLoS One. 2011 Apr 18;6(4):e18911. doi: 10.1371/journal.pone.0018911 (PMC3078932; doi:10.1371/journal.pone.0018911)
Supplement: Text S1 — (DOC) [file pone.0018911.s010.doc]

**Percid Divergence Dates**

Our analysis recovered a percid clade consisting of the North American Etheostominae and a Eurasian clade combining Luciopercinae and Percinae. The basal position of *Perca fluviatilis* within the Eurasian clade and the inferred date (21.77-45.16 Ma) for its divergence is in concordance with the earliest *P. fluviatilis* fossils from the Miocene (26 Ma) [29]. In addition, our date estimate based on six constraints for the split between North American and Eurasian percids (node T, 28.54-54.71 Ma) agrees well with laurasian vicariance following the breakup of landbridges between North America and Europe 50-40 Ma [30]. This shows that clades closely related to notothenioids were dated reliably. All acanthomorph divergence date estimates are summarized in Table S2.
