# Supplementary material for: On the Origin and Trigger of the Notothenioid Adaptive Radiation
Source: PLoS One. 2011 Apr 18;6(4):e18911. doi: 10.1371/journal.pone.0018911 (PMC3078932; doi:10.1371/journal.pone.0018911)
Supplement: Text S2 — (DOC) [file pone.0018911.s011.doc]

**Sample Collection**

Specimens of 14 notothenioid and 53 non-notothenioid acanthomorph fish species were acquired during field expeditions or from local dealers. We included putative sister groups of notothenioids [1, 2], acanthopterygiid relatives, as well as paracanthopterygiid outgroups [3, 4]. We deliberately constrained the number of notothenioid species to reduce sampling bias while at the same time including representatives of each family. Muscle or fin tissue samples were taken from all specimens and preserved in 95% ethanol. Genomic DNA was extracted by proteinase K digestion followed by sodium chloride extraction and ethanol precipitation.
