# Supplementary material for: On the Origin and Trigger of the Notothenioid Adaptive Radiation
Source: PLoS One. 2011 Apr 18;6(4):e18911. doi: 10.1371/journal.pone.0018911 (PMC3078932; doi:10.1371/journal.pone.0018911)
Supplement: Text S3 — (DOC) [file pone.0018911.s012.doc]

**Marker Selection**

Phylogenetic analyses were based on sequences of four nuclear and two mitochondrial genes. Out of ten nuclear markers developed by genome comparison strategy [5], we chose myh6, Ptr, ENC1, and tbr1 in order to include slow, intermediate and fast-evolving genes. A similar approach was taken for mitochondrial markers: preliminary phylogenetic analyses were performed with 53 published full mitochondrial genomes of acanthomorph fishes [3, 4], including three notothenioid species [6]. Hereby, species selection focused on clades G and H of [3], as percids had been suggested as a notothenioid sister group [1]. Mitogenomic sequences were aligned using MUSCLE v3.6 [7] and ProAlign 0.5a3 [8], and preliminary phylogenies were produced with with RAxML-VI-HPC [9]. Phylogenies of single mitochondrial genes were compared with the phylogeny of full mitogenomes (excluding dloop, ND6, and tRNAGlu sequences due to their poor phylogenetic performance [3]) to evaluate the suitability of every marker. We found ND4 [10] and cytochrome *b* (cyt *b*) sequences to reproduce the full mitogenome phylgeny better than other mitochondrial markers of comparable length, and thus included these markers in our analysis (Fig. S1).
