# Supplementary material for: On the Origin and Trigger of the Notothenioid Adaptive Radiation
Source: PLoS One. 2011 Apr 18;6(4):e18911. doi: 10.1371/journal.pone.0018911 (PMC3078932; doi:10.1371/journal.pone.0018911)
Supplement: Text S4 — (DOC) [file pone.0018911.s013.doc]

**Primers, PCR, and Sequencing**

Nuclear markers were PCR amplified using primer pairs myh6_F459/myh6_R1325, ptr_F458/ptr_R1248, enc1_F88/enc1_R975, and tbr1_F86/tbr1_R820 [5], and the following cycling conditions: 94˚C 2 min, [94˚C 30 s, 51-60˚C 30 s, 72˚C 1 min] × 11-37 cycles, 72˚C 7 min. Annealing temperatures were 51˚C (myh6), 55˚C (Ptr), 60-55˚C (ENC1), and 57˚C (tbr1), and the heating cycle was repeated 37 (myh6), 35 (Ptr), 46 (ENC1), or 32 (tbr1) times. ENC1 annealing temperature was reduced by 0.5˚C per cycle over the first eleven cycles. The following, newly developed primers were used for amplification of mitochondrial genes: NotND4_F416 (CGN TGA GGD AAY CAR RCA GAA CG), NotND4_R1137 (TTD GGD AGD GGD GGD AGD GC), NotCytBf (GGC AAG CCT CCG AAA AAC CCA CCC), L14724t, H15915t (AAC CYY CGR TRT CCG GYT TAC AAG AC), and H15915n (AAC CTY CGG CCT CCG GTT TAC AAG AC). Of these, NotCytBf was designed to bind at position 4 of notothenioid cyt *b* sequences, since the binding site of traditional cyt *b* forward primers [11] has been rearranged in notothenioids of the Antarctic Clade [12]. L14724t, H15915t, and H15915n were modified from the commonly used cyt *b* primers L14724 and H15915 [11] to account for variation found in teleost [3, 4], and notothenioid cyt *b* sequences [6]. NotND4_F416 and NotND4_R1137 design was based on mitogenomic alignments of acantomorph fishes [3, 4]. Cycling conditions for mitochondrial sequences were 95˚C 3 min, [96˚C 15 s, 54˚C 30 s, 68˚C 30 s] × 37 cycles, 72˚C 7 min for ND4, and 94˚C 2 min, [94˚C 30 s, 67-63˚C 30 s, 72˚C 1 min] × 39 cycles, 72˚C 7 min for cyt *b*, whereby, again the annealing temperature was decreased by 0.5˚C per cycle over the first nine cycles. PCR products were purified with ExoSAP-IT (USB), and in some instances using GenElute PCR Clean-Up and Gel Extraction kits (Sigma-Aldrich). Cycle sequencing was performed using the BigDye Terminator v3.1 Cycle Sequencing kit (Applied Biosystems). Sequencing reactions contained 0.5 μM primer, 1.0 μl BigDye Terminator Reaction Mix (Applied Biosystems), and 1.0-3.0 μl purified DNA in a total volume of 8 μl. The following profile was used for cycle sequencing 94˚C 1 min, [94˚C 10 s, 52˚C 20 s, 60˚C 4 min] × 25 cycles. Sequence base calls were carried out with CodonCode Aligner 2.0.6 (CodonCode), and verified by eye.
