# Supplementary material for: On the Origin and Trigger of the Notothenioid Adaptive Radiation
Source: PLoS One. 2011 Apr 18;6(4):e18911. doi: 10.1371/journal.pone.0018911 (PMC3078932; doi:10.1371/journal.pone.0018911)
Supplement: Text S5 — (DOC) [file pone.0018911.s014.doc]

**Sequence Editing and Alignment**

Forward and reverse sequences were assembled into contigs in CodonCode Aligner 2.0.6. The dataset was complemented with additional sequences from GenBank, Ensemble (www.ensembl.org), and Genoscope (www.genoscope.cns.fr) to a total of 83 acanthomorph fish species (Tables S1-S2). For every gene, sequences were aligned using MAFFT v6.717b [13]. Alignments were trimmed to start and end with codon triplets, and uninformative insertions were removed. Alignment lengths were 627 bp (ND4), 1140 bp (cyt *b*), 783 bp (ENC1), 705 bp (myh6), 702 bp (Ptr), and 642 bp (tbr1). In addition to alignments for every single marker, we produced a full concatenated alignment (‘full’, 4599 bp, 6.53% missing data) as well as separate concatenations for mitochondrial (‘mit123’) and nuclear (‘nuc123’) gene sequences. Furthermore, alignments containing all first and second codon positions of mitochondrial (‘mit12’) and nuclear (‘nuc12’) sequences, and alignments containing only third codon positions (‘mit3’ and ‘nuc3’) were generated to allow codon position-based model selection and phylogenetic reconstruction.
