# Supplementary material for: On the Origin and Trigger of the Notothenioid Adaptive Radiation
Source: PLoS One. 2011 Apr 18;6(4):e18911. doi: 10.1371/journal.pone.0018911 (PMC3078932; doi:10.1371/journal.pone.0018911)
Supplement: Text S6 — (DOC) [file pone.0018911.s015.doc]

**Model Selection and Phylogenetic Reconstruction**

Likelihood scores were computed for 88 substitution models on the basis of Maximum Likelihood (ML) optimized phylogenies, as implemented in jModelTest v0.1.1 [14, 15]. Best-fitting models of nucleotide substitution were selected for every alignment according to the Bayesian Information Criterion (BIC) [16]. Selected models were GTR+I+Γ (ND4 and mit123), TIM3+I+Γ (cyt *b* and ENC1),TPM2+I+Γ (myh6), HKY+I+Γ (Ptr, tbr1, and mit12) TVM+Γ (mit3), TPM1uf+I+Γ (nuc123), K80+I+Γ (nuc12), and GTR+Γ (nuc3). Maximum Likelihood phylogenetic inference was performed using a partition-enabled version of GARLI, GARLI-PART v0.97 [17], as well as RAxML v7.26 [9]. For GARLI-PART analyses, alignments mit12, mit3, nuc12, and nuc3 were employed as a concatenated set and the four respective codon position-based models were implemented. Five paracanthopterygian species (*Polymixia japonica*, *Polymixia nobilis*, *Aphredoderus sayanus*, *Gadus morhua*, and *Zeus faber* [18]) were defined as outgroups and 10 independent run replicates were performed. Runs were set to terminate after a maximum of 5 million generations, or alternatively after 10 000 generations without significant (p = 0.01) improvement of scoring topology. To assess node support, 100 nonparametric bootstrap (BS) replicates were run and summarized in a majority rule consensus tree in PAUP* v4.0a110 [19]. A combined bootstrap and ML search was conducted in RAxML, using 1000 rapid bootstrap inferences [20]. Substitution models selected by BIC were not available in RAxML, and the GTRCAT model [21] was chosen instead for all partitions. Given the placement of the serranid species *Serranus atricauda* in a clade containing Percidae and Trachinoidei in both GARLI-PART and RAxML analyses (Fig. 1), all phylogenetic inferences were repeated excluding this species. Removal of *Serranus atricauda* from the dataset affected tree topology only in the position of *Antigonia capros* (now basal to all Lophiiformes and Tetraodontiformes), and on average improved BS support by 1.03% (GARLI-PARTS) and 0.13% (RAxML) per node (Table S3).
