# Supplementary material for: On the Origin and Trigger of the Notothenioid Adaptive Radiation
Source: PLoS One. 2011 Apr 18;6(4):e18911. doi: 10.1371/journal.pone.0018911 (PMC3078932; doi:10.1371/journal.pone.0018911)
Supplement: Text S7 — (DOC) [file pone.0018911.s016.doc]

**Fossils Used to Constrain Node Ages**

We used eight fossil, and two phylogeographic constraints in order to estimate acanthomorph divergence dates. Here, we describe and reference each fossil constraint. Unless otherwise noted, all information is taken from [22].

Node A (split between Polymixiidae and both Zeidae and Gadidae): This split is constrained by the earliest polymixiid fossils including *Berycopsis elegans* Dixon, 1850, and *Homonotichthys* spp. from the Lower Chalk of Kent and Sussex, England, UK, and *Omosoma tselfatensis* Gaudant, 1978, and *Omosomopsis simum* [23] from Jebel Tselfat, Morocco, all skeletal and dated to the Cenomanian. We thus constrained node A with a wide lognormal prior (offset: 93.5 Ma, mean: 126.6 Ma, 95% cumulative prior probability (CPP): 328.6-98.2 Ma).

Node B (split between Zeidae and Gadidae): A putative minimum age for the divergence of Zeidae and Gadidae is given by an undescribed ?zeid from the Fur Formation, NW Jutland, Denmark, and by *Palaeocyttus princeps* Gaudant, 1978 from Laveiras, Portugal, both skeletal and dated to the Thanetian. However, the zeid assignment of *P. princeps* is questionable. A lognormal prior was applied (offset: 55.8 Ma, mean: 75.9 Ma, 95% CPP: 198.4-58.6 Ma).

Node C (divergence of Percomorpha): The minimum age derives from the oldest percomorph, *Plectocretacicus clarae* Sorbini, 1979, a skeletal fossil from the Lithographic Limestones of Hakel, Libanon, which has been dated to ≥ 96.9 Ma [24]. We assume that the diversification of Percomorpha postdates the earliest euteleost record, represented by *e.g.* *Tischlingerichthys viohli* Arratia, 1997, from the Solnhofen Limestone, Germany, that has an maximum age of 150.8 ± 0.1 Ma [24]. We thus applied a uniform prior between 150.9-96.9 Ma.

Node D (split between Gempylidae and Scombridae): The earliest gempylid record is provided by *Eutrichiurides orpiensis* Leriche, 1906, a skeletal fossil from the Montian Phosphates of Morocco, and an isolated teeth of *E. africanus* Dartevelle and Casier, 1949, from Landana, Angola. Both fossils have been dated to the Danian. The earliest scombrid fossils are *Landanichthys lusitanicus* Dartevelle and Casier, 1949, *L. moutai* Dartevelle and Casier, 1949, and *Sphyraenodus multidentatus* Dartevelle and Casier, 1949 of the scombrid tribe Scomberomorini, which are also from Landana, Angola, and are also assigned a Danian age. We applied a lognormal prior (offset: 61.7 Ma, mean 73.9 Ma, 95% CPP: 148.2-63.4 Ma). Note that the scombrid record traditionally includes Istiophoridae and Xiphiorhynchidae, and thus represents not necessarily a monophyletic group [25].

Node E (split between African and Neotropical cichlids): Phylogeographic constraint, see Materials and Methods for details.

Node F (split between *Labrus* and both *Ctenolabrus* and *Tautogolabrus* within Labridae): Phylogeographic constraint, see Materials and Methods. Following Hanel *et al.* [26], we assume that Labrini diversified subsequent to the closure of the seaway between the Indopacific and the Mediterranean. A minimum age for Labrus is provided by *Labrus agassizi* Heckel from St. Magarethen, Austria, which has been dated to the Upper Badenium (14.0 Ma). We applied a uniform prior between 20.5-14.0 Ma.

Node G (split between Gasterosteiformes and Tetraodontiformes): As the oldest percomorph, *Plectocretacicus clarae* Sorbini, 1979, is considered a stem-Tetraodontiform, we applied the same prior as for node C (Divergence of Percomorpha). See above.

Node H (split between Chaunacidae and Melanocetidae): A minimum age for this split is provided by the earliest chaunacid, *Chaunax semiangulatus* Stinton, 1978, an otolith fossil from the Barton Formation, Hampshire, England, UK, that is dated to the Bartonian. We thus applied a lognormal prior (offset: 37.2 Ma, mean: 44.6 Ma, CPP: 89.7-38.2 Ma).

Node I (split between Balistidae and Monacanthidae): This split is constrained by the oldest monacanthid fossil, *Amanses sulcifer* Stinton, 1966, an otolith fossil from the London Clay Formation, England, UK, dated to the Ypresian. A lognormal prior was applied (offset: 48.6 Ma, mean: 60.8 Ma, CPP 135.1-50.3 Ma).

Node J (divergence of Tetraodontidae): The earliest tetraodontid is *Archaeotetraodon winterbottomi* Tyler and Bannikov, 1994, a skeletal fossil from the Pshekhsky Horizon, in the lower part of the Maikop Formation of the north Caucasus, Russia, which has been dated to ≥ 32.25 Ma (Benton & Donoghue 2007). Following Benton & Donoghue (2007), we assume that the divergence of tetraodontids postdates the earliest fossil record of potential sister clades, *e.g.* the balistid *Moclaybalistes danekrus* Santini and Tyler, 2002, from the Fur Formation NW Jutland, Denmark, that has been dated to the base of the Eocene. Therefore, we applied a uniform prior for the divergence of Tetraodontidae between 56.0-32.25 Ma.
