# Supplementary material for: On the Origin and Trigger of the Notothenioid Adaptive Radiation
Source: PLoS One. 2011 Apr 18;6(4):e18911. doi: 10.1371/journal.pone.0018911 (PMC3078932; doi:10.1371/journal.pone.0018911)
Supplement: Text S8 — (DOC) [file pone.0018911.s017.doc]

**Applicability of Relaxed Molecular Clocks for Notothenioidei**

Acanthomorph divergence dates were estimated using an uncorrelated lognormal relaxed molecular clock, as implemented in the software BEAST. While this approach allows for independent rate variation among branches, it does not account explicitly for parameters that have been shown to influence substitution rates, including temperature, metabolic rate, body size, and generation time [27, 28]. Thus, it could be argued that application of a naive uncorrelated lognormal relaxed clock introduces systematic bias to divergence date estimates of Antarctic ectotherms. As a result of particularly low substitution rates, short branches of Antarctic notothenioids could be misinterpreted as recent divergences. However, we see little evidence for unusually low substitution rates in Antarctic notothenioids. In fact, one of the lowest notothenioid substitution rates is inferred for *Pseudaphritis urvillii*, a basal riverine species occurring in southern Australia, rather than for high Antarctic species (Fig. S4). Similarly, ML branch lengths of Antarctic notothenioids are consistently longer, instead of shorter, than those of non-Antarctic notothenioids and closely related riverine percids from the northern hemisphere (Fig. 1). We thus conclude that systematic bias by temperature regime does not affect notothenioid divergence dates and that the use of an uncorrelated lognormal relaxed molecular clock appropriately accounts for occurring rate variation.

**References**

1. Dettaï A, Lecointre G (2004) In search of notothenioid (Teleostei) relatives. Antarct Sci 16: 71-85.

2. Smith WL, Craig MT (2007) Casting the percomorph net widely: the importance of broad taxonomic sampling in the search for the placement of serranid and percid fishes. Copeia 2007: 35-55.

3. Kawahara R, et al. (2008) Interrelationships of the 11 gasterosteiform families (sticklebacks, pipefishes, and their relatives): A new perspective based on whole mitogenome sequences from 75 higher teleosts. Mol Phylogenet Evol 46: 224-236.

4. Mabuchi K, Miya M, Azuma Y, Nishida M (2007) Independent evolution of the specialized pharyngeal jaw apparatus in cichlid and labrid fishes. BMC Evol Biol 7:10.

5. Li C, Ortí G, Zhang G, Lu G (2007) A practical approach to phylogenomics: the phylogeny of ray-finned fish (Actinopterygii) as a case study. BMC Evol Biol 7: 44.

6. Papetti C, Liò P, Rüber L, Patarnello T, Zardoya R (2007) Antarctic fish mitochondrial genomes lack ND6 gene. J Mol Evol 65: 519-528.

7. Edgar RC (2004) MUSCLE: multiple sequence alignment with high accuracy and high throughput. Nucl Acids Res 32: 1792-1797.

8. Löytynoja A, Milinkovitch MC (2003) A hidden Markov model for progressive multiple alignment. Bioinformatics 19: 1505-1513.

9. Stamatakis A (2006) RAxML-VI-HPC: maximum likelihood-based phylogenetic analyses with thousands of taxa and mixed models. Bioinformatics 22: 2688-2690.

10. Miya M, Saitoh K, Wood R, Nishida M, Mayden RL (2006) New primers for amplifying and sequencing the mitochondrial ND4/ND5 gene region of the Cypriniformes (Actinopterygii: Ostariophysi). Ichthyol Res 53: 75-81.

11. Meyer A, Kocher TD, Basasibwaki P, Wilson AC (1990) Monophyletic origin of Lake Victoria cichlid fishes suggested by mitochondrial DNA sequences. Nature 347: 550-553.

12. Zhuang X, Cheng C-HC (2010) ND6 gene “lost” and found: evolution of mitochondrial gene rearrangement in Antarctic notothenioids. Mol Biol Evol 27: 1391-1403.

13. Katoh K, Toh H (2008) Recent developments in the MAFFT multiple sequence alignment program. Brief Bioinform 9: 286-298.

14. Posada D (2008) jModelTest: phylogenetic model averaging. Mol Biol Evol 25: 1253-1256.

15. Guindon S, Gascuel O (2003) A simple, fast, and accurate algorithm to estimate large phylogenies by maximum likelihood. Syst Biol 52: 696-704.

16. Schwarz G (1978) Estimating the dimension of a model. Ann Stat 6: 461-464.

17. Zwickl DJ (2006) Genetic algorithm approaches for the phylogenetic analysis of large biological sequence datasets under the maximum likelihood criterion. Ph.D. dissertation. Univ. of Texas at Austin.

18. Miya M, et al. (2003) Major patterns of higher teleostean phylogenies: a new perspective based on 100 complete mitochondrial DNA sequences. Mol Phylogenet Evol 26: 121-138.

19. Swofford DL (2003) PAUP*. Phylogenetic analysis using parsimony (*and other methods). Version 4. Sinauer Associates, Sunderland, MA.

20. Stamatakis A, Hoover P, Rougemont J (2008) A rapid bootstrap algorithm for the RAxML web servers. Syst Biol 57: 758-771.

21. Stamatakis A (2006) Phylogenetic models of rate heterogeneity: a high performance computing perspective. Proceedings of IPDPS2006, High Performance Computational Biology Workshop, Rhodos, Greece.

22. Patterson C (1993) Osteichthyes: Teleostei. In: Benton MJ, editor. The fossil record 2. London: Chapman & Hall. pp. 621-655.

23. Arambourg C (1954) Les poissons crétacés du Jebel Tselfat (Maroc). Notes et Mémoires du Service Géologique, Maroc 118: 1-188.

24. Benton MJ, Donoghue P (2007) Paleontological evidence to date the tree of life. Mol Biol Evol 24: 26-53.

25. Orrell TM, Collette BB, Johnson GD (2006) Molecular data support separate scombroid and xiphioid clades. B Mar Sci 79: 505-519.

26. Hanel R, Westneat MW, Sturmbauer C (2002) Phylogenetic relationships, evolution of broodcare behavior, and geographic speciation in the wrasse tribe Labrini. J Mol Evol 55: 776-789.

27. Estabrook GF, Smith GR, Dowling TE (2007) Body mass and temperature influence rates of mitochondrial DNA evolution in North American cyprinid fish. Evolution 61: 1176-1187.

28. Nabholz B, Glemin S, Galtier N (2008) Strong variations of mitochondrial mutation rate across mammals - the longevity hypothesis. Mol Biol Evol 25: 120-130.

29. Carney JP, Dick TA (2000) The historical ecology of yellow perch (*Perca flavenscens* [Mitchill]) and their parasites. J Biogeogr 27: 1337-1347.

30. Milne RI, Abbott RJ (2002) The origin and evolution of Tertiary relict floras. In: Callow JA, editor. Advances in Botanical Research Vol. 38. London: Academic Press. pp 298-301.

31. Nelson (2006) Fishes of the World. Fourth Edition. New Jersey: John Wiley & Sons, Inc. 601 p.

32. Murray AM (2001) The oldest fossil cichlids (Teleostei: Perciformes): indication of a 45 million-year-old species flock. Proc R Soc B 268: 679-684.

33. Malabarba MC, Malabarba LR, Del Papa C (2010) *Gymnogeophagus eocenicus*, n. sp (Perciformes: Cichlidae), an Eocene cichlid from the Lumbrera Formation in Argentina. J Vertebr Palaeontol 30: 341-350.

34. Santini F, Tyler JC (2003) A phylogeny of the families of fossil and extant tetraodontiform fishes (Acanthomorpha, Tetraodontiformes), Upper Cretaceous to Recent. Zool J Linn Soc-Lond 139: 565-617.

35. Eastman JT, Grande L (1991) Late Eocene gadiform (Teleostei) skull from Seymour Island, Antarctic Peninsula. Antarct Sci 3: 87-95.

36. Balushkin AV (1994) Fossil notothenioid, and not gadiform, fish *Proeleginops grandeastmanorum* gen. nov. sp. nov. (Perciformes, Notothenioidei, Eleginopidae) from the late Eocene found in Seymour Island (Antarctica). J Ichthyol 34: 298-307.
